# Effects of preoperative topical levofloxacin on conjunctival microbiome in patients undergoing intravitreal injections

**DOI:** 10.1371/journal.pone.0320785

**Published:** 2025-03-31

**Authors:** Jakkrit Juhong, Wijak Kongwattananon, Nuntachai Surawatsatien, Nathapon Treewipanon, Tanittha Chatsuwan

**Affiliations:** 1 School of Medicine, Walailak University, Nakhon Si Thammarat, Thailand; 2 Department of Ophthalmology, Walailak University Hospital, Walailak University, Nakhon Si Thammarat, Thailand; 3 Department of Ophthalmology, Faculty of Medicine, Center of Excellence in Retina, Chulalongkorn University, Bangkok, Thailand; 4 Department of Ophthalmology, King Chulalongkorn Memorial Hospital, Thai Red Cross Society, Bangkok, Thailand; 5 Department of ophthalmology, Faculty of Medicine, Naresuan University, Phitsanulok, Thailand; 6 Department of Microbiology, Faculty of Medicine, Chulalongkorn University, Bangkok, Thailand; 7 Center of Excellence in Antimicrobial Resistance and Stewardship, Faculty of Medicine, Chulalongkorn University, Bangkok, Thailand; National Taiwan University Hospital, Taiwan

## Abstract

**Purpose:**

Endophthalmitis is a serious eye infection that can occur after intravitreal injections. Topical antibiotics are frequently used as a preventative measure, but their impact on the conjunctival microbiome is not fully understood.

**Methods:**

Conjunctival swabs were collected from 33 eyes of 33 patients undergoing intravitreal injections, both before and after a 3-day course of prophylactic topical levofloxacin 0.5%. Conjunctival microbiome analysis was conducted using 16S rRNA sequencing on the Illumina MiSeq platform. Bioinformatics processing identified unique amplicon sequence variants (ASVs) to evaluate microbial diversity and community composition. Alpha and beta diversity indices were analyzed.

**Results:**

Topical levofloxacin treatment resulted in no significant change in alpha diversity indices, including Shannon index, Chao1, Shannon, PD whole tree, and observed ASVs, indicating stable microbial richness and evenness. In contrast, beta diversity analysis, assessed through Bray-Curtis dissimilarity, revealed significant differences in microbial composition between pre- and post-treatment samples. These changes included a decrease in the abundance of *Staphylococcus* and *Bacillus* species and an increase in the abundance of *Streptococcus, Haemophilus, and Neisseria.*

**Conclusion:**

Although prophylactic topical levofloxacin was found to alter the conjunctival microbiome and showed inconsistent effects on the abundance of pathogenic bacteria, its clinical effectiveness as a preventative measure against endophthalmitis remains inconclusive. Further studies are needed to clarify its role in infection prevention.

## Introduction

Post-intravitreal injection (IVI) endophthalmitis is a rare but potentially sight-threatening complication, with incidence rates ranging from 0.0018% after a single injection to 0.5% following multiple injections [1]. Among preventive measures, the application of topical povidone-iodine is widely regarded as the most critical step in reducing this risk [2]. The use of prophylactic topical antibiotics before IVIs, however, remains controversial, with evidence indicating limited efficacy in preventing endophthalmitis. A large Japanese study involving 147,440 IVIs found no significant difference in endophthalmitis rates between antibiotic-treated and untreated groups [3], corroborating data from the Retina Consultants of Houston, which reported similar findings in 90,339 IVIs [4]. Current guidelines emphasize povidone-iodine antisepsis as the primary evidence-based strategy for preventing endophthalmitis [2,5]. Despite these recommendations, the use of antibiotics persists in some regions, such as Japan, where 97.2% of practitioners prescribe them, contrasting with US-based survey, where only 21% report using pre-injection antibiotics [3,6]. Additionally, a UK-based survey emphasized the variability in antibiotic use and noted that guidelines discourage routine prophylaxis due to insufficient evidence of effectiveness and concerns about resistance [7]. Previous culture-based studies have reported that the use of topical fluoroquinolones significantly reduces conjunctival bacterial growth [8–10]. However, their direct impact on preventing endophthalmitis remains uncertain.

The ocular surface microbiome, composed of a diverse array of microorganisms, plays a critical role in maintaining ocular health by protecting against pathogenic organisms and modulating immune responses [11]. It is recognized as a potential source of infection in post-IVI endophthalmitis [12].

Although recent studies using microbial sequencing to investigate the effects of topical antibiotics on the conjunctival microbiome have been limited, understanding these changes is crucial, as they can influence both the risk of infection and the health of the ocular surface. In this study, we used 16S rRNA gene sequencing to explore how the conjunctival microbiome is altered following the application of prophylactic topical antibiotics in individuals undergoing IVI.

## Methods

The study was approved by the Institutional Review Board, Faculty of Medicine, Chulalongkorn University (IRB No. 0597/65 and COA No.1522/2022), and all methods was conducted in accordance with the Declaration of Helsinki. Informed consent was obtained from all subjects for all examinations and procedures.

### Subjects and study design

The study was conducted at King Chulalongkorn Memorial Hospital, Bangkok, Thailand. Treatment-naïve patients over 18 years old with only unilateral retinal diseases requiring IVI of anti-VEGF were included. The exclusion criteria were as follows: subjects with a known allergy to fluoroquinolone drugs; those with active ocular surface diseases (e.g., acute conjunctivitis, corneal ulcer, severe dry eye, Stevens-Johnson Syndrome); those who has used any ocular medication, systemic antibiotics, steroids, or immunosuppressive drug within the past three months; those with a history of ocular trauma or surgery within the past three months; and those with monocular vision or functional monocular vision.

A sample size of 33 eyes was calculated using a formula for comparing two dependent means, with the standard deviation derived from a previous study [13, 14]. Subjects diagnosed with retinal diseases were scheduled for intravitreal injections of anti-VEGFs at the Department of Ophthalmology, King Chulalongkorn Memorial Hospital, between December 2022 and May 2023. Ocular surface samples were obtained from the treated eye in two sessions: pre-antibiotic (Pre-ATB) and post-antibiotic (Post-ATB). Conjunctival swabbing, culturing, DNA extraction, and 16S rRNA sequencing were performed by masked investigators (N.T.).

### Sample collection and DNA storage

On the first day of recruitment, Pre-ATB conjunctival swabs were collected from the treatment eyes. Three minutes after application of topical anesthesia (0.5% Tetracaine Hydrochloride Solution, Alcon®). A sterile cotton swab was then gently swept across the inferior conjunctival surface from the nasal to the temporal side three times, rotating the swab 360 ° with each pass to ensure thorough collection while avoiding any trauma to the conjunctival tissue. The swab was then placed in a DNase-free tube containing a DNA/ RNA shield solution (Zymo, Irvine, CA, USA) and transported to the laboratory. A sterile cotton swab was placed in the transport medium without being used for the swab, serving as a negative control. The samples were stored at -20 ° C and DNA was extracted in one week. Following this, subjects were prescribed levofloxacin 0.5% eye drops (Santen, Japan) to be used four times daily for three days before the intravitreal injections. On the day of injection, conjunctival swabs were collected again from the same eye using the same protocol. Before injection, aseptic technique was strictly applied throughout the procedure. A 10% povidone-iodine solution was applied to the periocular skin, upper and lower eyelids, and eyelid margin, while a 5% povidone-iodine solution was instilled onto the conjunctiva as eye drops. The intravitreal injections were performed using a pre-filled 30-gauge needle.

#### Next generation sequencing analysis.

Bacterial DNA was extracted by the QIAamp DNA Microbiome Kit (QIAGEN, Germany). The extracted DNA was amplified using the REPLI-g Mini kit (QIAGEN, Germany). The amplification of V3-V4 variable region of the 16S rRNA gene was determined using 341F and 805R primers and sparQ HiFi PCR Master Mix (Quantabio, USA). Cluster generation and 250-bp paired-end read sequencing were performed on an Illumina MiSeq (Illumina, USA) at the Omics Sciences and Bioinformatics Center (Chulalongkorn University, Bangkok, Thailand).

### Statistical analysis

#### Bioinformatics analyses.

Microbiome bioinformatics were performed with DADA2 v1.16.0 pipeline (https://benjjneb.github.io/dada2/). The DADA2 pipeline describes microbial diversity and community structures using unique amplicon sequence variants (ASVs)[15]. ASVs which have a total frequency of less than 55 reads and reads of mitochondria and chloroplast will be filtered out. Microbial taxa were classified from Silva version 138 as a reference database [16]. Alpha diversity index (Observed ASVs, Chao1, Shannon, and PD whole tree) was computed using DADA2 software. For Beta diversity, non-metric multidimensional scaling (NMDS) based on Bray-Curtis dissimilarity and principal coordinate analysis (PCoA) were plotted from Phyloseq data. Linear discriminant analysis effect size (LEfSe) [17] was performed to identify the bacterial biomarkers.

#### Data analysis.

Demographic data were analyzed using descriptive statistics. Pairwise comparison of alpha diversity (Observed ASVs, Chao1, Shannon, and PD whole tree) was calculated using ANOVA test. Permutational multivariate analysis of variance (PERMANOVA) [18] was performed to evaluate the significant differences for beta diversity among groups. Moreover, the Kruskal-Wallis sum-rank test was also used in LEfSe analysis to identify bacterial biomarkers that differed significantly in abundant taxon between groups. *P* < 0.05 was considered statistically significant.

## Results

A total of 33 eyes from 33 patients were included in this study. The mean age was 67.6 ±  11.6 years old (range 45-88 years old), and 43% were male. Retinal disease diagnoses included diabetic macular edema (DME) in 15 patients (45.5%), polypoidal choroidal vasculopathy (PCV) in 8 (24.2%), age-related macular degeneration (AMD) in 6 (18.2%), myopic choroidal neovascularization (CNV) in 2 (6.1%) and retinal vein occlusion (RVO) in 2 (6.1%), respectively. None of the patients developed post-IVI endophthalmitis.

### Next-generation sequencing analysis

Illumina sequencing of 16S rRNA genes produced a total of 4,320,354 reads. Following quality data processing, 3,653,042 high-quality reads were retained. On average, each sample yielded 65,459 reads, with a range from 5,162 to 157,695. A total of 2,862 ASVs were identified. The estimated saturation of microbial richness across all samples was approximately 3,241 sequencing depths, as indicated by rarefaction curves, which plateaued around 3,000 sequencing depths.

### Taxonomic composition of conjunctival microbiome community

**Fig 1:**
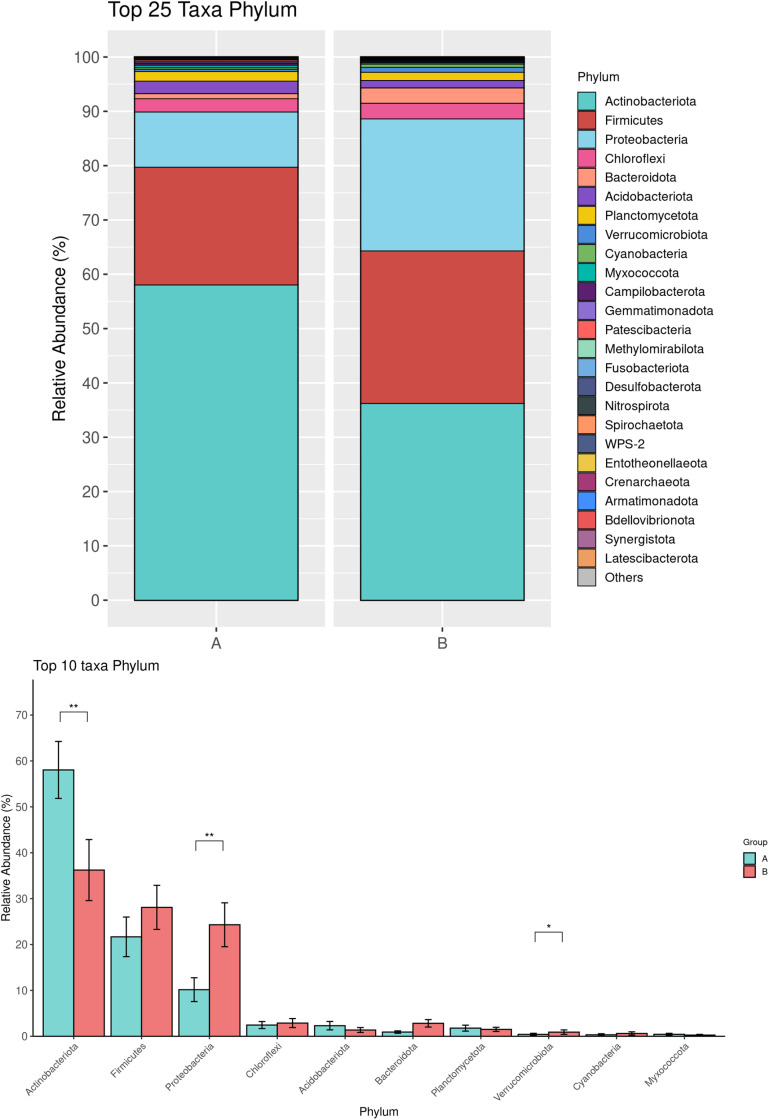
Top 25 bacterial taxa at the phylum level of the ocular surface microbiome in Pre-ATB (A) and Post-ATB (B) groups (top). Comparison of the top 10 phyla between the Pre-ATB (A) and Post-ATB (B) groups (bottom). (*p value <  0.05; **p value <  0.001; ***p value <  0.0001).

**Fig 2:**
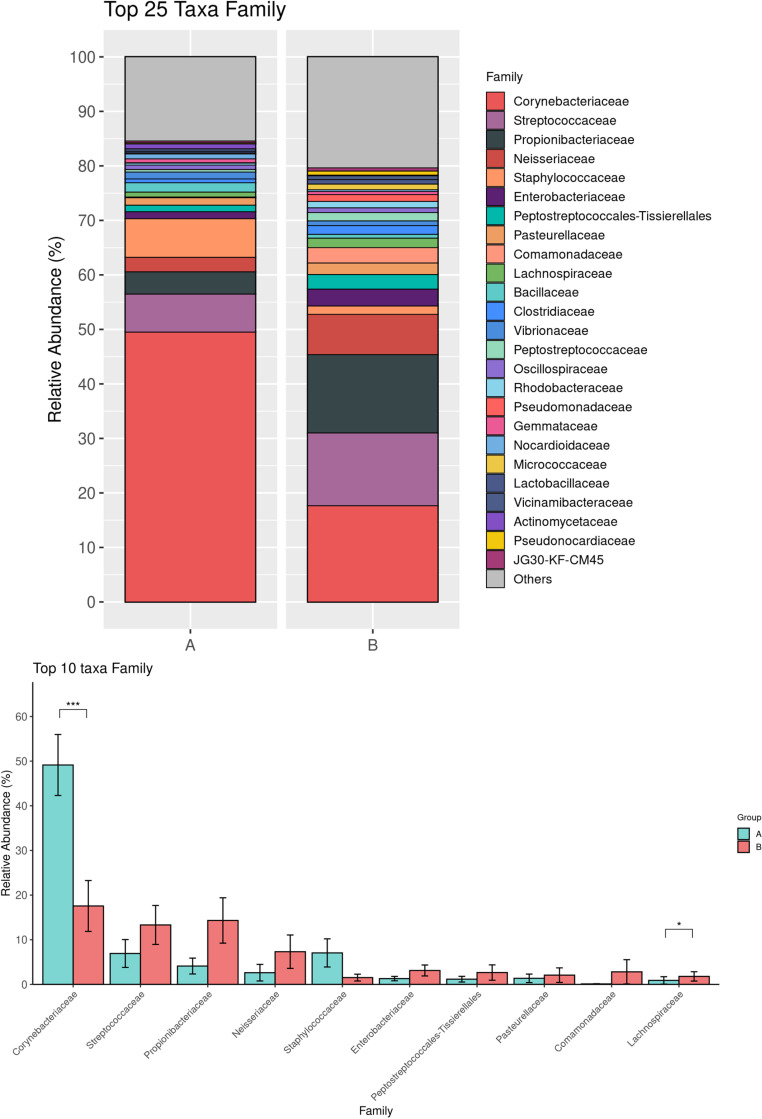
Top 25 bacterial taxa at the family level of the ocular surface microbiome in Pre-ATB (A) and Post-ATB (B) groups (top). Comparison of the top 10 family between the Pre-ATB (A) and Post-ATB (B) groups (bottom). (*p value <  0.05; **p value <  0.001; ***p value <  0.0001).

**Fig 3:**
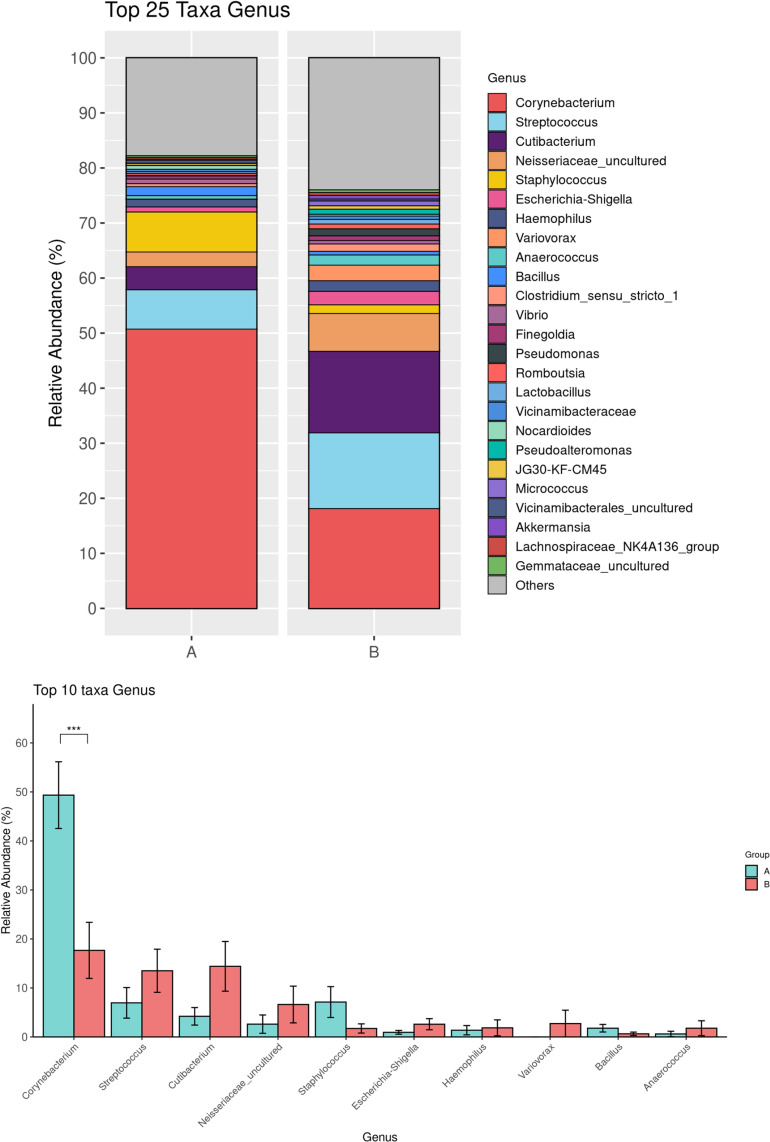
Top 25 bacterial taxa at the genus level of the ocular surface microbiome in Pre-ATB (A) and Post-ATB (B) groups (top). Comparison of the top 10 genus between the Pre-ATB (A) and Post-ATB (B) groups (bottom). (*p value <  0.05; **p value <  0.001; ***p value <  0.0001).

A total of 36 bacterial phyla, 40 classes, 376 families, and 720 genera were identified. The most abundant phylum in both Pre-ATB and Post-ATB samples was Actinobacteriota, comprising 58.03% and 36.22% of the bacterial composition, respectively. This was followed by Firmicutes (21.67% in Pre-ATB and 28.08% in Post-ATB), Proteobacteria (10.16% and 24.30%), and Chloroflexi (2.45% and 2.88%) (Fig 1). The abundance of Actinobacteria was significantly lower in Post-ATB compared to Pre-ATB (36.22% vs. 58.04%, p =  0.009) (Fig 1). In contrast, Proteobacteria was significantly more abundant in Post-ATB compared to Pre-ATB (24.30% vs. 10.16%, p =  0.005), as was Verrucomicrobiota (0.9% vs. 0.42%, p =  0.02) (Fig 1). At the family level, Corynebacteriaceae was significantly more abundant in Pre-ATB than in Post-ATB (49.14% vs. 17.57%, p =  0.002) (Fig 2). Similarly, at the genus level, *Corynebacterium* showed a significant decrease in abundance in Pre-ATB than in Post-ATB (49.34% vs. 17.66%, p =  0.0002) (Fig 3). Although *Staphylococcus* and *Bacillus* were less abundant in Post-ATB (1.73% and 2.97%, respectively) compared to Pre-ATB (7.12% and 1.78%, respectively), these differences were not statistically significant (p =  0.252 and p =  0.843, respectively) (Fig 3). *Streptococcus* showed an increased abundance in Post-ATB (13.5% vs. 6.95%, p =  0.072), though this difference also had no statistical significance (Fig 3).

**Fig 4:**
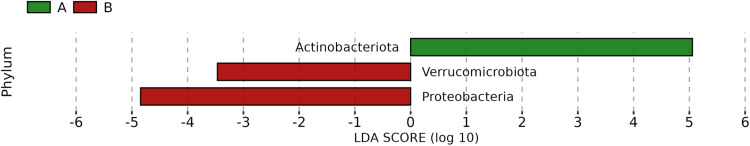
Linear discriminant analysis effect size (LEfSe) biomarker analysis showing biomarker phylum with significance differential abundance in Pre-ATB (A) and Post-ATB (B). Bacterial taxa with LDA scores greater than ± 2 were considered significant. LEfSe analysis.

**Fig 5:**
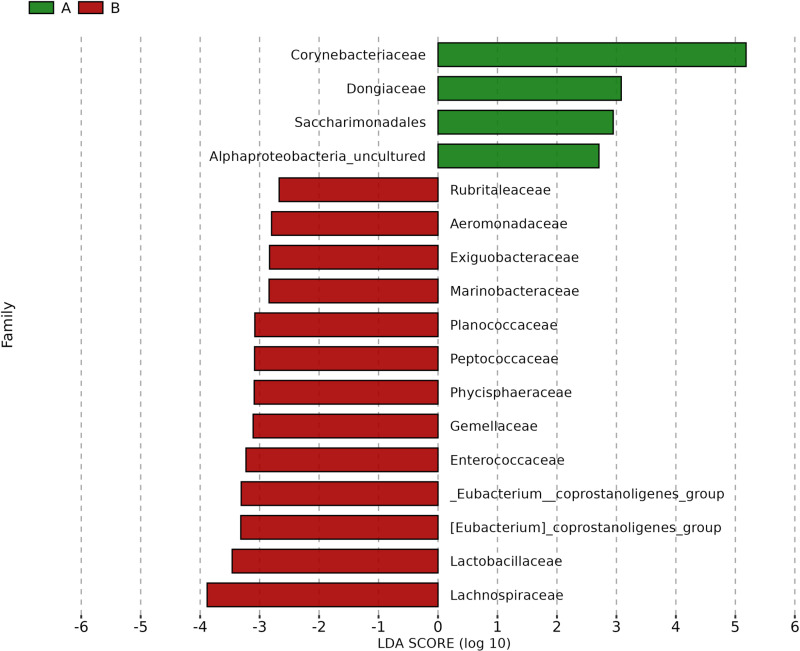
Linear discriminant analysis effect size (LEfSe) biomarker analysis showing biomarker family with significance differential abundance in Pre-ATB (A) and Post-ATB (B). Bacterial taxa with LDA scores greater than ± 2 were considered significant. LEfSe analysis.

**Fig 6:**
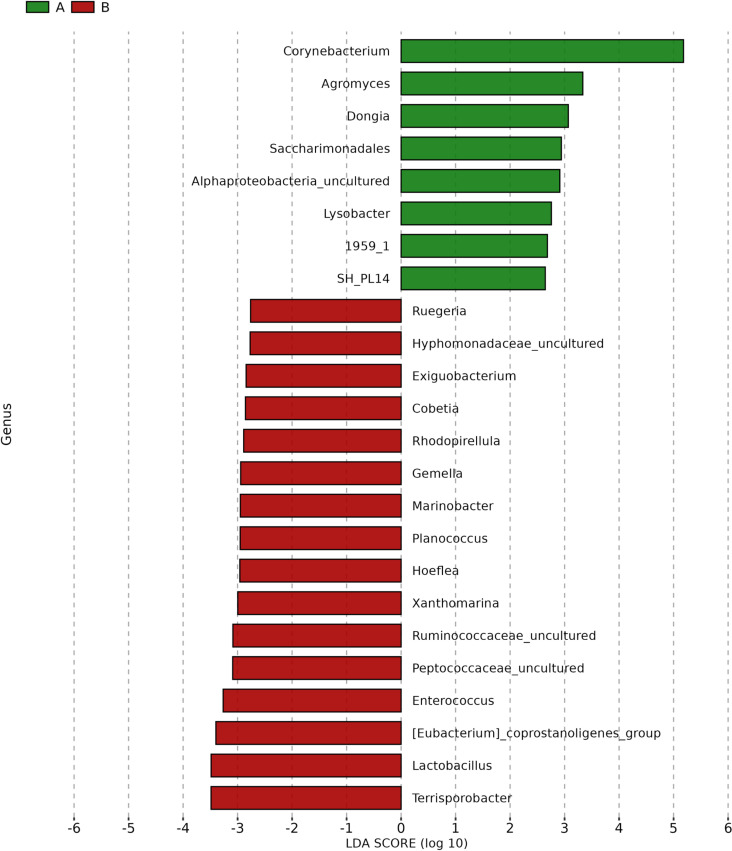
Linear discriminant analysis effect size (LEfSe) biomarker analysis showing biomarker genus with significance differential abundance in Pre-ATB (A) and Post-ATB (B). Bacterial taxa with LDA scores greater than ±2 were considered significant. LEfSe analysis.

**Fig 7:**
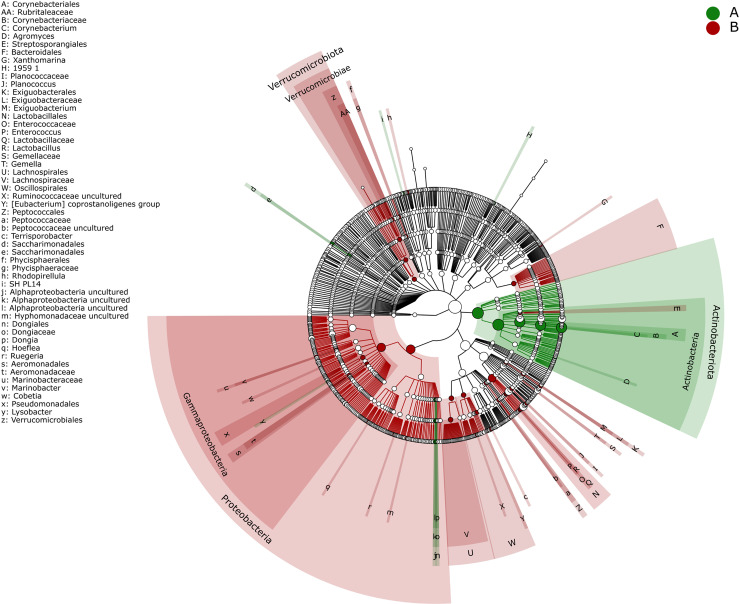
Linear discriminant analysis effect size (LEfSe) biomarker analysis yielded a cluster tree, with different colors representing different groups. Nodes of different colors represent microbial communities that played an important role in the same group. Colored circles represent biomarkers, and yellow nodes represent microbial groups that did not play an important role in any of the groups investigated. For concentric circles, moving from the center to the outside, circles represent the level of phylum, class, order, family and genus.

Linear Discriminant Analysis Effect Size (LEfSe) was applied to identify potential biomarkers distinguishing Pre-ATB and Post-ATB groups by evaluating significant variations in bacterial distribution. The bar chart displays the effect size, represented by LDA (Linear Discriminant Analysis), for taxa identified as significant in each group. Taxa with LDA scores greater than 2 were considered significant. The results are presented in Fig 4, Fig 5, Fig 6, and Fig 7.

#### Alpha-diversity.

**Fig 8:**
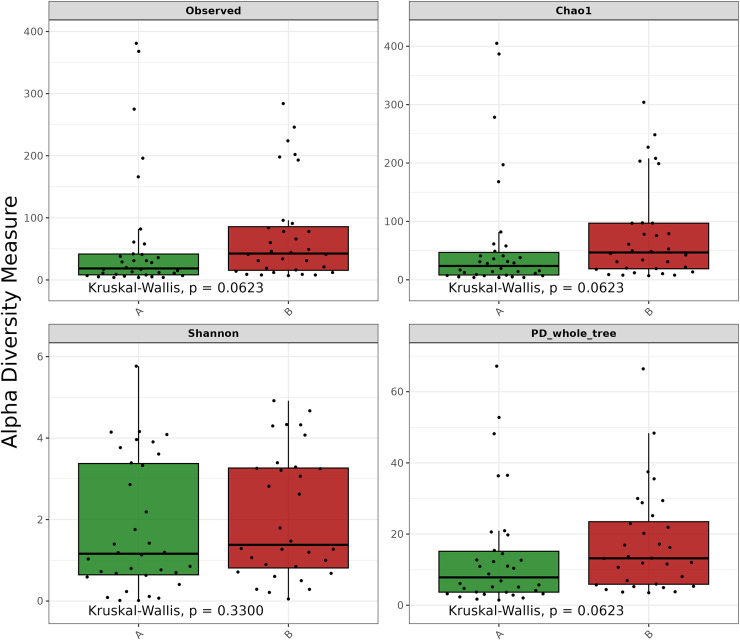
Alpha diversity boxplots: Observed ASVs, Chao1, Shannon, and phylogenetic diversity (PD) whole tree evaluation indexes were used to analyze the alpha diversity of all samples. From top to bottom, the five lines represent: minimum, first quartile, median, third quartile and maximum. (*p value <  0.05).

Alpha-diversity was assessed using observed ASVs, Chao1, Shannon, and phylogenetic diversity (PD) whole tree. No significant differences were found between the Pre-ATB and Post-ATB groups (p-value >  0.05). The box plots illustrating the alpha-diversity are shown in Fig 8.

#### Beta-diversity.

**Fig 9:**
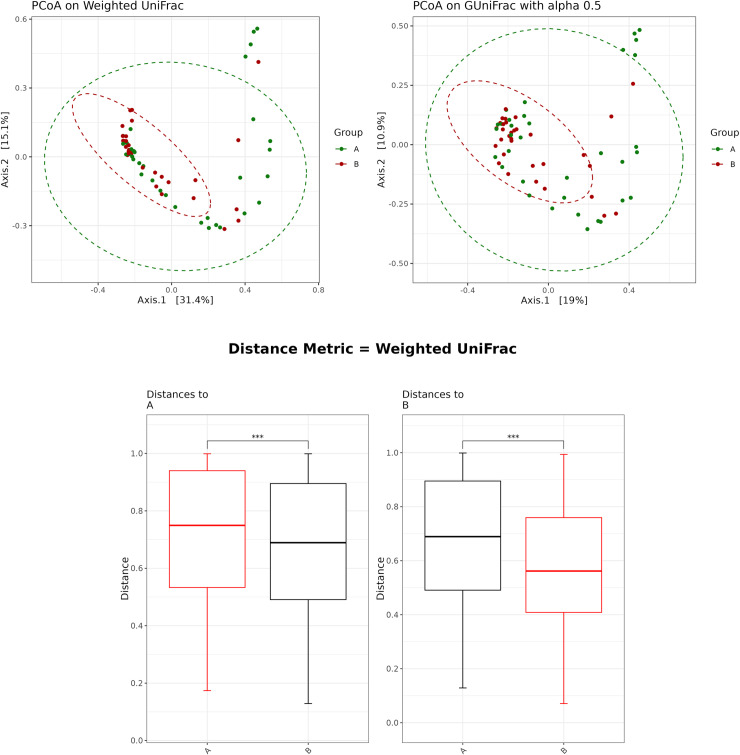
Beta-diversity between the Pre-ATB (green-dotted circle) and Post-ATB (red-dotted circle) by principal coordinate analysis (PCoA) plot based on weighted UniFrac and GUniFrac distance with comparison of ocular surface microbiome between groups (box plot). (**p value <  0.001; ***p value <  0.0001).

Principal coordinate analysis (PCoA) using weighted UniFrac and GUniFrac distances revealed a significant difference in the human ocular surface microbial communities between Pre-ATB and Post-ATB (PERMANOVA test; p <  0.05) (Fig 9). Pairwise analysis of the distance metrics further confirmed a significant difference between the two groups in the weighted UniFrac PCoA (p <  0.001) (Fig 9).

## Discussion

Endophthalmitis, a serious complication following IVI, is primarily caused by microorganisms introduced during the procedure. The most common pathogens include *Staphylococcus epidermidis, Staphylococcus aureus,* and *Streptococcus* species, along with gram-negative bacteria such as *Escherichia coli* and *Pseudomonas aeruginosa* [19, 20]. This study aimed to investigate the impact of topical levofloxacin administration on the conjunctival microbiome in eyes that received intravitreal injections. Levofloxacin eye drops were chosen for this study due to rapid onset of action and broad-spectrum antibacterial activity against both gram-positive and gram-negative organisms. The three-day regimen allows for sustained antibacterial activity without promoting significant bacterial resistance, which can occur with prolonged antibiotic use [10,21].

Before the use of topical, three predominant phyla identified in our samples were *Acinetobacter* (58.03%), *Firmicutes* (21.67%), and *Proteobacteria (*10.16%). These three were commonly reported in the earlier studies on the ocular microbiome using conjunctival swabs in healthy individual [22, 23]. However, our study shows a greater relative abundance of *Acinetobacter.* At the genus level, the most commonly identified ocular bacteria (defined as > 1% of all detected genera) were *Corynebacterium* (49.34%), *Staphylococcus* (7.12%), and *Streptococcus* (6.96%), which are considered potential core genera. However, certain genera, including *Acinetobacter*, *Pseudomonas*, and *Propionibacterium,* were less abundant compared to previous studies [24]. These differences could be related to variations in the composition of the conjunctival microbiome among the enrolled subjects and the method used for sample collection.

After the use of topical antibiotics, our study found no significant changes in alpha diversity metrics, including the Shannon index and species richness (p >  0.05). This stability suggests that levofloxacin does not cause a broad reduction in microbial diversity but instead exerts targeted effects. However, beta diversity analysis revealed significant shifts in microbial community composition (PERMANOVA, p <  0.05). Specifically, the relative abundance of *Staphylococcus* and *Bacillus* species decreased, while *Streptococcus* and gram-negative bacteria, such as *Haemophilus*, increased. These compositional changes align with known antibiotic susceptibility patterns. For example, a Korean study examining conjunctival flora in patients undergoing anterior segment surgery reported susceptibility rates of 78.7% for coagulase-negative staphylococci (CNS) and 94.6% for gram-negative isolates to topical levofloxacin [25]. The increased abundance of *Haemophilus*, despite its high susceptibility to levofloxacin, may reflect ecological shifts driven by the suppression of gram-positive bacteria. Such alterations in the microbiome could compromise the conjunctival barrier, potentially increasing susceptibility to pathogen proliferation during invasive procedures like intravitreal injections. These findings underscore the importance of judicious antibiotic selection to maintain microbial homeostasis and minimize the risk of complications.

In addition, we found the shifts in particular microbial populations after topical antibiotics. A distinct core composition was found *Marinobacteraceae, Gemellaceae, Exiguobacteraceae, Lachnospiraceae*, and *Rubritaleaceae* emerging as the dominant bacterial families (p <  0.05). Notably, none of these altered core microbiota have been associated with post-IVI endophthalmitis [19]. This altered composition significantly differs from the healthy ocular surface microbiome reported in previous studies [11,26,27]. Based on the results, we propose that levofloxacin may alter the microbial composition; however, its effectiveness in fully eradicating potentially pathogenic bacteria is variable, as certain bacteria may persist or appear more dominant after treatment due to shifts in community structure rather than actual proliferation.

There have been a few studies evaluating the effect of use of perioperative topical antibiotics on conjunctiva microbiomes. Ya-Guang Hu et al. reported that the use of levofloxacin eye drops in patients who had undergone multiple intravitreal injections resulted in a decrease in the diversity of the ocular surface microbiome [28]. They also observed changes in the composition, including a relative increase in the abundance of gram-negative bacteria, such as *Proteus.* Zhu et el. conducted a study comparing the conjunctival microbiome of patients with type 2 diabetes mellitus (T2DM) and non-diabetic controls undergoing cataract surgery [29]. After three days of preoperative topical levofloxacin, *Staphylococcus* was found to be significantly more abundant genus in the conjunctival microbiome of patients with T2DM compared to controls. However, no significant changes in the conjunctival microbiome were observed in the control group. The discrepancy between their findings and ours may be explained by several key factors. First, differences in study populations: While our study included a diverse group of patients undergoing IVI with various diagnoses, the Zhu et al. study specifically compared T2DM and non-diabetic patients undergoing cataract surgery [29]. This focus on T2DM patients likely reflects differences in baseline microbiome composition, as T2DM is known to predispose individuals to higher baseline levels of *Staphylococcus* and other opportunistic pathogens due to systemic dysbiosis. Second, variation in sample collection and antibiotic exposure: Although both studies utilized 16S rRNA sequencing to profile the conjunctival microbiome, our study collected samples after a three-day course of preoperative levofloxacin. In contrast, the Zhu et al. study involved a longer antibiotic regimen that included both pre- and postoperative levofloxacin treatment. This extended exposure may have selectively enriched *Staphylococcus* in the diabetic microbiome, which is inherently more vulnerable to dysbiosis and microbial shifts [29].

Previous clinical studies found a lack of effect regarding the use of topical antibiotic prophylaxis on the rate of endophthalmitis after IVI [30,31]. This may be due to several factors. First, endophthalmitis is a rare complication, and the low baseline incidence makes it challenging to detect statistically significant differences between those receiving prophylactic antibiotics and those who do not. Additionally, the widespread adoption of effective surgical techniques, stringent sterile protocols, and the use of antiseptics such as povidone-iodine may already reduce infection risk to such an extent that the added benefit of topical antibiotics becomes negligible. This finding was supported by a culture-based study. Following the use of three days of gatifloxacin eye drops prior to intravitreal injection, the rate of positive bacterial cultures decreased. However, this decrease was not observed with the use of povidone iodine [10].

The overuse of perioperative topical antibiotics raises concerns about bacterial resistance, which can diminish their effectiveness and potentially increase the risk of endophthalmitis. In a retrospective case-control study, the incidence of endophthalmitis following intravitreal injections increased despite the use of topical antibiotic prophylaxis, which was also associated with a higher incidence of antibiotic resistance among culture-positive cases [30]. Yin et al. also found that the repeated use of topical moxifloxacin following intravitreal injections significantly increases antibiotic resistance on the ocular surface [32].

Our study is limited due to the small sample size. Thus, we could not perform our analysis based on patients’ preexisting conditions such as diabetes and aging, as these may have different baseline conjunctival microbiomes and may yield varying results with the topical antibiotics. Further prospective studies with varying durations and coverage spectra of topical antibiotics may provide deeper insights. Additionally, microbiome analysis alone cannot directly identify antibiotic-resistant strains. The use of culture-based methods would allow for the identification of live bacteria and enable antibiotic susceptibility testing, addressing the question of whether the use of topical antibiotics could potentially lead to the selection of resistant microbial strains.

Another limitation of this study is the lack of quantitative measures, such as quantitative-PCR (qPCR), to assess absolute bacterial loads. This limitation may have led to an under or overestimation of bacterial abundance in post-antibiotic samples when analyzing relative abundances. Future studies incorporating quantitative methods would help provide a more accurate assessment of bacterial load changes.

In conclusion, our study demonstrated that the use of levofloxacin eye drops for prophylaxis against post-intravitreal injection endophthalmitis alters the composition of the conjunctival microbiome. However, the effectiveness of this antibiotic regime in reducing the risk of endophthalmitis remains uncertain. Further research is needed to develop tailored antibiotic strategies that optimize preoperative prophylaxis while minimizing the risk of postoperative infections.

## Supporting information

S1 File Data analysed during this study. (XLSX)

## References

[1] Israilevich RN, Mansour H, Patel SN, Garg SJ, Klufas MA, Yonekawa Y, et al. Risk of endophthalmitis based on cumulative number of anti-VEGF intravitreal injections. Ophthalmology. 2024;131(6):667–73. doi: 10.1016/j.ophtha.2023.12.033 38182029

[2] Avery RL, Bakri SJ, Blumenkranz MS, Brucker AJ, Cunningham ET Jr, DʼAmico DJ, et al. Intravitreal injection technique and monitoring: updated guidelines of an expert panel. Retina. 2014;34(12):S1–18. doi: 10.1097/IAE.0000000000000399 25489719

[3] Morioka M, Takamura Y, Nagai K, Yoshida S, Mori J, Takeuchi M, et al. Incidence of endophthalmitis after intravitreal injection of an anti-VEGF agent with or without topical antibiotics. Sci Rep. 2020;10(1):22122. doi: 10.1038/s41598-020-79377-w 33335269 PMC7747565

[4] Li AL, Wykoff CC, Wang R, Chen E, Benz MS, Fish RH, et al. Endophthalmitis after intravitreal injection: role of prophylactic topical ophthalmic antibiotics. Retina. 2016;36(7):1349–56. doi: 10.1097/IAE.0000000000000901 26655622

[5] Lai TYY, Liu S, Das S, Lam DSC. Intravitreal injection--Technique and safety. Asia Pac J Ophthalmol (Phila). 2015;4(6):321–8. doi: 10.1097/APO.0000000000000146 26649760

[6] Chaturvedi R, Wannamaker KW, Riviere PJ, Khanani AM, Wykoff CC, Chao DL. Real-world trends in intravitreal injection practices among american retina specialists. Ophthalmol Retina. 2019;3(8):656–62. doi: 10.1016/j.oret.2019.03.023 31133544 PMC6684447

[7] Samia-Aly E, Cassels-Brown A, Morris DS, Stancliffe R, Somner JEA. A survey of UK practice patterns in the delivery of intravitreal injections. Ophthalmic Physiol Opt. 2015;35(4):450–4. doi: 10.1111/opo.12217 26094833

[8] Miño de Kaspar H, Kreutzer TC, Aguirre-Romo I, Ta CN, Dudichum J, Bayrhof M, et al. A prospective randomized study to determine the efficacy of preoperative topical levofloxacin in reducing conjunctival bacterial flora. Am J Ophthalmol. 2008;145(1):136–42. doi: 10.1016/j.ajo.2007.08.031 17996212

[9] Celebi ARC, Onerci Celebi O. The effect of topical ocular moxifloxacin on conjunctival and nasal mucosal flora. Sci Rep. 2021;11(1):13782. doi: 10.1038/s41598-021-93233-5 34215812 PMC8253760

[10] Moss JM, Sanislo SR, Ta CN. A prospective randomized evaluation of topical gatifloxacin on conjunctival flora in patients undergoing intravitreal injections. Ophthalmology. 2009;116(8):1498–501. doi: 10.1016/j.ophtha.2009.02.024 19501409

[11] Petrillo F, Pignataro D, Lavano MA, Santella B, Folliero V, Zannella C, et al. Current evidence on the ocular surface microbiota and related diseases. Microorganisms. 2020;8(7):1033. doi: 10.3390/microorganisms8071033 32668575 PMC7409318

[12] Speaker MG, Milch FA, Shah MK, Eisner W, Kreiswirth BN. Role of external bacterial flora in the pathogenesis of acute postoperative endophthalmitis. Ophthalmology. 1991;98(5):639–49; discussion 650. doi: 10.1016/s0161-6420(91)32239-5 2062496

[13] De Caro J, Ta CN, Ho HKV, Cabael L, Hu N, Sanislo SR, et al. Bacterial contamination of ocular surface and needles in patients undergoing intravitreal injections. Invest Ophthalmol Visual Sci. 2008;28(6):877–83. doi: 10.1097/iae.0b013e31816b31 18536606

[14] Ozkan J, Coroneo M, Sandbach J, Subedi D, Willcox M, Thomas T. Bacterial contamination of intravitreal needles by the ocular surface microbiome. Ocul Surf. 2021;19 169–75. doi: 10.1016/j.jtos.2020.05.010 32497656

[15] Callahan BJ, McMurdie PJ, Rosen MJ, Han AW, Johnson AJA, Holmes SP. DADA2: High-resolution sample inference from Illumina amplicon data. Nat Methods. 2016;13(7):581–3. doi: 10.1038/nmeth.3869 27214047 PMC4927377

[16] Quast C, Pruesse E, Yilmaz P, Gerken J, Schweer T, Yarza P, et al. The SILVA ribosomal RNA gene database project: improved data processing and web-based tools. Nucleic Acids Res. 2013;41(Database issue):D590-6. doi: 10.1093/nar/gks1219 23193283 PMC3531112

[17] Segata N, Izard J, Waldron L, Gevers D, Miropolsky L, Garrett WS, et al. Metagenomic biomarker discovery and explanation. Genome Biol. 2011;12(6):R60. doi: 10.1186/gb-2011-12-6-r60 21702898 PMC3218848

[18] Anderson MJ. Permutational multivariate analysis of variance (PERMANOVA). Wiley StatsRef: Statistics Reference Online; 2017. p. 1-15.

[19] Labardini CP, Blumenthal EZ. Causative pathogens in endophthalmitis after intravitreal injection of anti-vascular endothelial growth factor agents. Rambam Maimonides Med J. 2018;9(4):1–6. doi: 10.5041/RMMJ.10348 30180932 PMC6185999

[20] Sutter FKP, Gillies MC. Pseudo-endophthalmitis after intravitreal injection of triamcinolone. Br J Ophthalmol. 2003;87(8):972–4. doi: 10.1136/bjo.87.8.972 12881337 PMC1771815

[21] Li X, Liang X, Tang L, Zhang J, Shen L, Su G, et al. Optimal duration for the use of 0.5% levofloxacineye drops before vitreoretinal surgery. Asia Pac J Ophthalmol (Phila). 2017;6(1):40–4. doi: 10.22608/APO.2015197 28161927

[22] Huang Y, Yang B, Li W. Defining the normal core microbiome of conjunctival microbial communities. Clin Microbiol Infect. 2016;22(7):643.e7-643.e12. doi: 10.1016/j.cmi.2016.04.008 27102141

[23] Matysiak A, Kabza M, Karolak JA, Jaworska MM, Rydzanicz M, Ploski R, et al. Characterization of ocular surface microbial profiles revealed discrepancies between conjunctival and corneal microbiota. Pathogens. 2021;10(4):405. doi: 10.3390/pathogens10040405 33808469 PMC8067172

[24] Delbeke H, Younas S, Casteels I, Joossens M. Current knowledge on the human eye microbiome: a systematic review of available amplicon and metagenomic sequencing data. Acta Ophthalmol. 2021;99(1):16–25. doi: 10.1111/aos.14508 32602257

[25] Park SH, Lim J-A, Choi J-S, Kim K-A, Joo C-K. The resistance patterns of normal ocular bacterial flora to 4 fluoroquinolone antibiotics. Cornea. 2009;28(1):68–72. doi: 10.1097/ICO.0b013e318182259b 19092409

[26] Zhou Y, Holland MJ, Makalo P, Joof H, Roberts CH, Mabey DC, et al. The conjunctival microbiome in health and trachomatous disease: a case control study. Genome Med. 2014;6(11):99. doi: 10.1186/s13073-014-0099-x 25484919 PMC4256740

[27] Miller D, Iovieno A. The role of microbial flora on the ocular surface. Curr Opin Allergy Clin Immunol. 2009;9(5):466–70. doi: 10.1097/ACI.0b013e3283303e1b 19620859

[28] Hu Y-G, Wu Q, Li T-H, Sui F, Zhang M, Zhang Z, et al. Effects of perioperative managements on ocular surface microbiota in intravitreal injection patients. Int J Ophthalmol. 2022;15(2):248–54. doi: 10.18240/ijo.2022.02.09 35186684 PMC8818460

[29] Zhu X, Wei L, Rong X, Zhang Y, Zhang Q, Wen X, et al. Conjunctival microbiota in patients with Type 2 diabetes mellitus and influences of perioperative use of topical levofloxacin in ocular surgery. Front Med (Lausanne). 2021;8:605639. doi: 10.3389/fmed.2021.605639 33889581 PMC8055849

[30] Storey P, Dollin M, Pitcher J, Reddy S, Vojtko J, Vander J, et al. The role of topical antibiotic prophylaxis to prevent endophthalmitis after intravitreal injection. Ophthalmology. 2014;121(1):283–9. doi: 10.1016/j.ophtha.2013.08.037 24144453

[31] Bhatt SS, Stepien KE, Joshi K. Prophylactic antibiotic use after intravitreal injection: effect on endophthalmitis rate. Retina. 2011;31(10):2032–6. doi: 10.1097/IAE.0b013e31820f4b4f 21659941 PMC4459136

[32] Yin VT, Weisbrod DJ, Eng KT, Schwartz C, Kohly R, Mandelcorn E, et al. Antibiotic resistance of ocular surface flora with repeated use of a topical antibiotic after intravitreal injection. JAMA Ophthalmol. 2013;131(4):456–61. doi: 10.1001/jamaophthalmol.2013.2379 23430175

